# Mono-(Ni, Au) and Bimetallic (Ni-Au) Nanoparticles-Loaded ZnAlO Mixed Oxides as Sunlight-Driven Photocatalysts for Environmental Remediation

**DOI:** 10.3390/molecules30153249

**Published:** 2025-08-02

**Authors:** Monica Pavel, Liubovi Cretu, Catalin Negrila, Daniela C. Culita, Anca Vasile, Razvan State, Ioan Balint, Florica Papa

**Affiliations:** 1 “Ilie Murgulescu” Institute of Physical Chemistry of the Romanian Academy, 202 Spl. Independentei, 060021 Bucharest, Romania; mpavel@icf.ro (M.P.); lcretu@icf.ro (L.C.); danaculita@yahoo.co.uk (D.C.C.); avasile@icf.ro (A.V.); rstate@icf.ro (R.S.); 2 National Institute of Material Physics, P.O. Box MG 7, 077125 Magurele, Romania; catalin.negrila@infim.ro

**Keywords:** nanoparticles, mixed oxides, bimetallic nanostructures, photocatalysis, bisphenol A, water remediation

## Abstract

A facile and versatile strategy to obtain NPs@ZnAlO nanocomposite materials, comprising controlled-size nanoparticles (NPs) within a ZnAlO matrix is reported. The mono-(Au, Ni) and bimetallic (Ni-Au) NPs serving as an active phase were prepared by the polyol-alkaline method, while the ZnAlO support was obtained via the thermal decomposition of its corresponding layered double hydroxide (LDH) precursors. X-ray diffraction (XRD) patterns confirmed the successful fabrication of the nanocomposites, including the synthesis of the metallic NPs, the formation of LDH-like structure, and the subsequent transformation to ZnO phase upon LDH calcination. The obtained nanostructures confirmed the nanoplate-like morphology inherited from the original LDH precursors, which tended to aggregate after the addition of gold NPs. According to the UV-Vis spectroscopy, loading NPs onto the ZnAlO support enhanced the light absorption and reduced the band gap energy. ATR-DRIFT spectroscopy, H_2_-TPR measurements, and XPS analysis provided information about the functional groups, surface composition, and reducibility of the materials. The catalytic performance of the developed nanostructures was evaluated by the photodegradation of bisphenol A (BPA), under simulated solar irradiation. The conversion of BPA over the bimetallic Ni-Au@ZnAlO reached up to 95% after 180 min of irradiation, exceeding the monometallic Ni@ZnAlO and Au@ZnAlO catalysts. Its enhanced activity was correlated with good dispersion of the bimetals, narrower band gap, and efficient charge carrier separation of the photo-induced e^−^/h^+^ pairs.

## 1. Introduction

Environmental degradation and the climate crisis represent two major challenges that require immediate action. Among the main contributors to ecological problems, plastic waste is a threat to ecosystems and human health due to improper discarding. Without measures, the annual flow of marine plastic trash is expected to grow by 2040 to 29 million metric tons [1]. According to the Organization for Economic Co-operation and Development (OECD) report, in 2019, only 9% of plastic waste was successfully recycled while 22% was mismanaged [2]. Many harmful substances originating from different types of plastic materials easily leach into water bodies (e.g., oceans, rivers, drinking water, groundwater) due to their disintegration [3].

Bisphenol A (BPA) is classified as one of the most pervasive emerging contaminants due to its multiple detrimental effects on human health and other organisms. This is a widely used chemical in the plastics industry; however, its severe toxicity induces adverse effects on reproduction, growth, and development [4,5]. In December 2024, the European Commission (EC) bans the use of Bisphenol A in food contact materials due to the European Food Safety Authority’s (EFSA) latest risk assessment [6]. In this way, numerous studies have been conducted on the development of effective BPA removal technologies, including ion exchange [7], ozonation [8], physicochemical and biological treatments [9], adsorption processes [10], etc. Among the available strategies, photocatalytic degradation is one of the most encouraging methods due to its effectiveness, mineralization of pollutants, and low cost of operation [11].

From a catalytic perspective, mixed oxides are more effective materials outperforming the individual oxides due to their larger surface area, porosity, and good stability [12]. So far, various synthesis methods of oxide compounds with controllable properties have been reported [13,14,15,16]. Layered double hydroxides (LDHs), also known as hydrotalcite-type anionic clays, are lamellar materials with outstanding physicochemical properties with applications in electronics, adsorbents, plastic, agriculture, catalysis, photocatalysis, and more [17]. These compounds are chemically expressed by the formula [M^2+^_1−x_M^3+^_x_(OH)_2_]^x+^[A^m−^_x/m_]^x−^·nH_2_O, where M^2+^ and M^3+^ are divalent and trivalent metal cations, A^n−^ is an anion, and 2 ≤ (1 − x)/x ≤ 4. The synthesis method of the LDHs is usually simple, eco-friendly, and cost-effective. Thermal decomposition of LDH precursors leads to their corresponding mixed metal oxides with distinctive properties such as high specific surface area, good stability and cation homogeneity [18].

One promising route to boost the photocatalytic activity of LDHs-derived mixed oxides is to decorate their surface with well-controlled nanoparticles (NPs). Due to the unique features of the NPs (e.g., size, shape), applications in environmental remediation, sensors, catalysis, the medical industry, agriculture, electronics, the automotive industry, energy storage, the food industry, and pharmaceuticals are well-recognized [19]. Noble metals (Pd, Pt, Au, etc.)-based catalysts are effective systems for pollution control reactions [20]. Fine and well-defined Pt, Pt–Cu, and Pt–Ag nanoparticles protected with PVP have been successfully synthesized by the alkaline polyol method [21] and supported on TiO_2_ and Al_2_O_3_. The above-stated catalysts were evaluated for the reduction in nitrates demonstrating high selectivity to nitrogen in the aqueous phase [22,23,24]. Sandulescu et al. [25] studied the TiO_2_ and noble metals (Ag, Au, Pt)-modified TiO_2_ catalysts for the photo-oxidation of phenol under sunlight irradiation. The metal-loaded TiO_2_ enhanced the photocatalytic process acting as visible absorbers and facilitating charge separation, resulting in the reduction in adsorbed oxygen (O_2_) to superoxide (O_2_^•−^). The superoxide radical mildly oxidized phenol into an oxygenated product. In a parallel process, hydroxyl radicals (•OH) yielded by TiO_2_ led to the mineralization of phenol pollutant. Among the non-platinum group metals, nickel is by far an excellent choice for various catalytic and photocatalytic processes due to its relatively low cost, low levels of toxicity, high activity, and potential usage in green chemistry [26]. On the other hand, gold-supported catalysts confer distinctive electrochemical properties and remarkable stability against corrosion being valorized for both industrial and environmental applications [27]. In this way, bimetallic nanoparticles offer advantages over single-metals catalysts due to the synergistic effect between the components leading to higher reaction rates, selectivity and stability [28].

Herein, a facile approach for designing effective nanocomposites that comprise uniformly sized nanoparticles (e.g., Ni, Au, and Ni-Au) dispersed over a homogeneous ZnAlO matrix was developed. The samples were characterized by multiple techniques to highlight their physicochemical properties. The efficiency of the NPs@ZnAlO nanocomposites was investigated for the photodegradation of Bisphenol A (BPA) under simulated solar light (SSL) irradiation. The Ni-Au@ZnAlO catalyst demonstrated the highest photocatalytic performance, achieving 95% BPA photodegradation, exceeding the monometallic-containing catalysts. The obtained Ni-Au@ZnAlO catalyst could be a promising material for environmental applications or other related processes.

## 2. Results and Discussion

### 2.1. Structural Analyses

**Figure 1:**
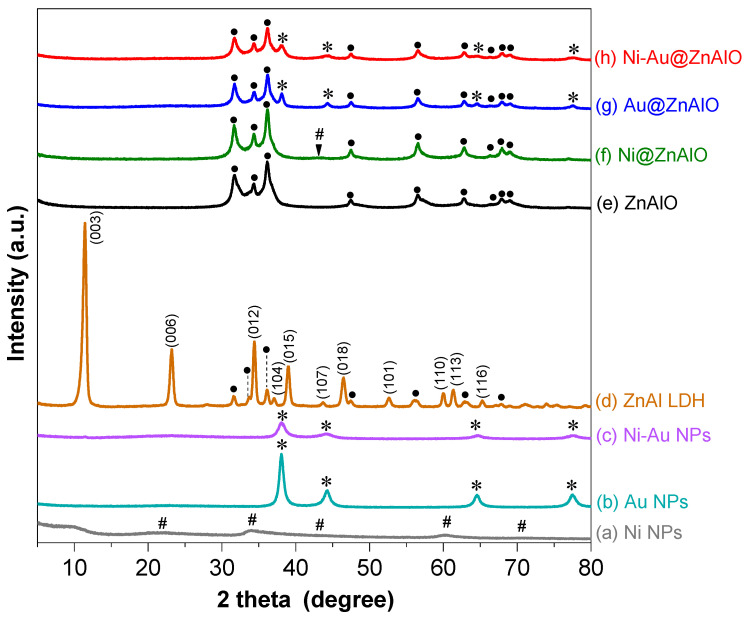
The XRD patterns of samples: (**a**) Ni NPs; (**b**) Au NPs; (**c**) Ni-Au NPs; (**d**) ZnAl LDH precursor; (**e**) ZnAlO mixed oxides; (**f**) Ni@ZnAlO; (**g**) Au@ZnAlO; and (**h**) Ni-Au@ZnAlO. Symbols #—Ni; *—Au; and •—ZnO in round brackets are given the Miller index for the LDH phase.

The crystalline structures of Ni, Au, Ni-Au nanoparticles (NPs), ZnAl LDH, ZnAlO, and NPs@ZnAlO were analyzed by X-ray diffraction (XRD). The XRD pattern of the NiNPs sample (Figure 1a) shows broadened peaks observed in the range of 20–71° and is attributed to the small size of nickel’s crystallites. This broadening occurs due to a limited number of repeating atomic planes, which reduces the coherence length and increases uncertainty in diffraction angles. Also, the intensity of the diffraction lines may decrease because fewer atoms contribute to the constructive interference. Strain or defects within the nanoparticles can cause slight shifts in peak positions, reflecting changes in lattice parameters. The research study conducted by Sun et al. [29] on molecular dynamics simulations demonstrated that small-diameter metallic nanoparticles tend to adopt an amorphous structure. The diffraction lines observed at 2θ of 38.01, 44.26, 64.50, and 77.58° were indexed to the (111), (200), (220), and (311) crystalline planes of Au (Figure 1b). Meanwhile, in the bimetallic Ni-Au NPs (Figure 1c), only the characteristic diffraction lines of gold were found.

The characteristic diffraction lines for ZnAl LDH sample (Figure 1d) were assigned to (003), (006), (101), (012), (104), (015), (107), (018), (101), (110), (113), and (116) planes of the layered double hydroxide (LDH) structure to rhombohedral 3R symmetry. The formula of the obtained LDH is Zn_0.71_Al_0.29_(OH)_2_(CO_3_)_0.145_·H_2_O (ICDD card no. 00-048-1021), while the *d*_003_ basal spacing (e.g., 0.77 nm) confirmed the carbonate anion intercalated LDH [30]. Simultaneously, the presence of a segregated ZnO phase in the LDH arrangement was evidenced. This observation is consistent with the literature data when the Zn/Al molar ratio is larger than 2 [31]. The position of the (110) reflection line allows the calculation of the lattice parameter ***a*** (2*d*_110_) related to the metal–metal distance in the hexagonal framework of ZnAl layered double hydroxide sheets. From the position of the (003) reflection, the ***c*** lattice parameter (3*d*_003_) representing the distance between interlayers was calculated. The obtained ***a*** and ***c*** values (0.31 nm and 2.32 nm, respectively) agree with LDH-based materials [32].

The calcination of the LDH precursor at 650 °C for 5 h (Figure 1e) produced a pure ZnO phase (ICDD card no. 01-071-6424). The obtained mixed metal oxide material was further used as support for the deposition of the mono-(Ni, Au), and bimetallic (Ni-Au) nanoparticles. Hence, for Ni@ZnAlO (Figure 1f), the diffraction lines of LDH disappeared and the zinc oxide phase was detected along with a flattened characteristic line of metallic Ni at 2 theta of 43.47°. The XRD patterns of both Au@ZnAlO and Ni-Au@ZnAlO nanocomposites (Figure 1g,h) show that the main diffraction lines of Au were simultaneously present with those of ZnO. The absence of the diffraction lines of nickel in the bimetallic NPs-containing ZnAlO was probably due to the amount of Ni loading, which was too low to be detected, or because of its homogeneous dispersion onto the ZnAlO support.

Additionally, the XRD quantitative analysis using the Reference Intensity Ratio (RIR) method allowed the determination of the content (%) of Au and Ni from the samples. Therefore, the amount of Au for the Au@ZnAlO sample was obtained to be 1.87%. Meanwhile, in the case of the Ni@ZnAlO catalyst, the value of Ni was found to be 1.36%. Concerning the Ni-Au@ZnAlO sample, it contained 0.96% Au, but the amount of nickel (Ni) content could not be determined for this sample.

The chemical composition of the mixed oxides, as free ZnAlO and NPs-loaded ones, determined by X-ray photoelectron spectroscopy (XPS) analysis, is given in Table 1. The results showed that the Zn^2+^/Al^3+^ molar ratio is close to the nominal value of 3 for all samples, which correlates with the values in solution for the ZnAlO mixed oxides derived from the corresponding LDH precursors.

The specific surface area (SSA) of the ZnAlO matrix was measured at 86 m^2^·g^−1^. With the presence of monometallic nanoparticles, the SSA decreased to 81 m^2^·g^−1^ and 78 m^2^·g^−1^ for Ni@ZnAlO and Au@ZnAlO, respectively. The addition of bimetallic nanoparticles led to a smaller specific surface area with a value of 74 m^2^·g^−1^ for Ni-Au@ZnAlO catalyst. 

### 2.2. Scanning Electron Microscopy (SEM)

**Figure 2:**
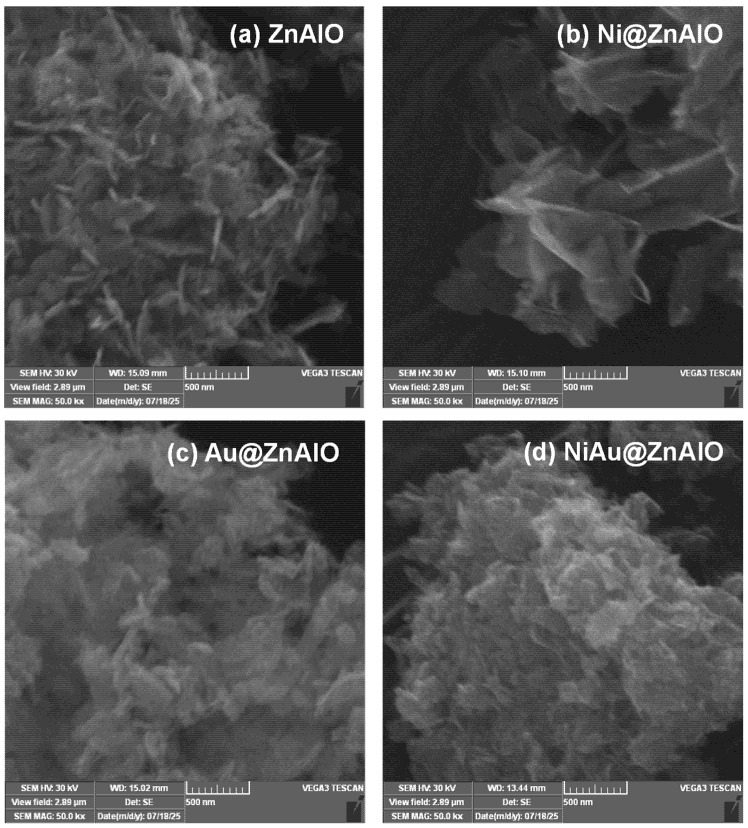
SEM images of (**a**) ZnAlO, (**b**) Ni@ZnAlO, (**c**) Au@ZnAlO, and (**d**) Ni-Au@ZnAlO catalysts.

The change in the microstructure of the ZnAlO and NPs-loaded ones was examined using scanning electron microscopy. All samples are characterized by a platelet-like morphology of varying sizes preserved from the original LDH precursors, which is more noticeable in the cases of ZnAlO and Ni@ZnAlO solids (Figure 2a,b). After the loading of gold nanoparticles, the samples tend to aggregate (Figure 2c), and a covering of small Ni NPs by larger Au NPs (Figure 2d) is possible. This observation confirms the absence of the nickel diffraction lines from the XRD pattern of the bimetallic-containing sample.

**Figure 3:**
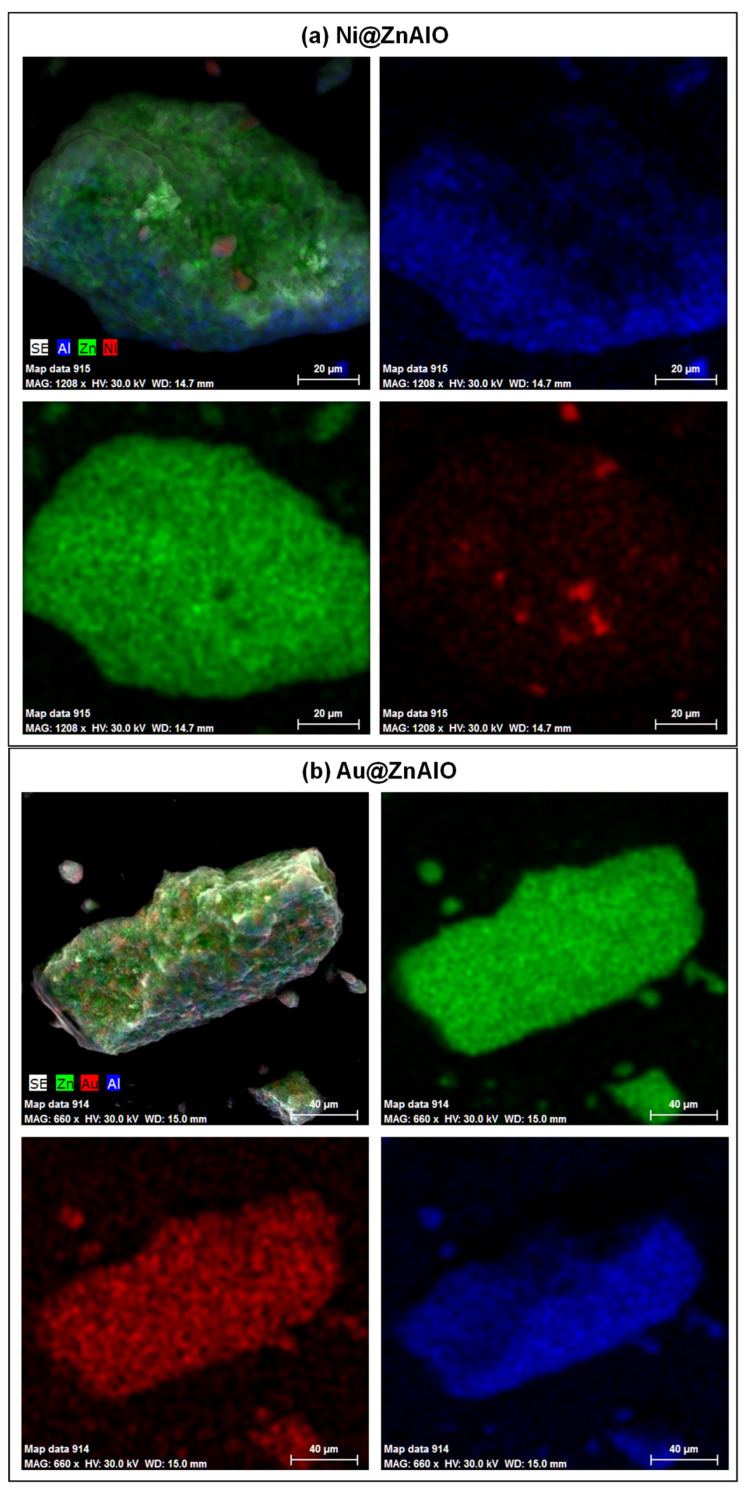

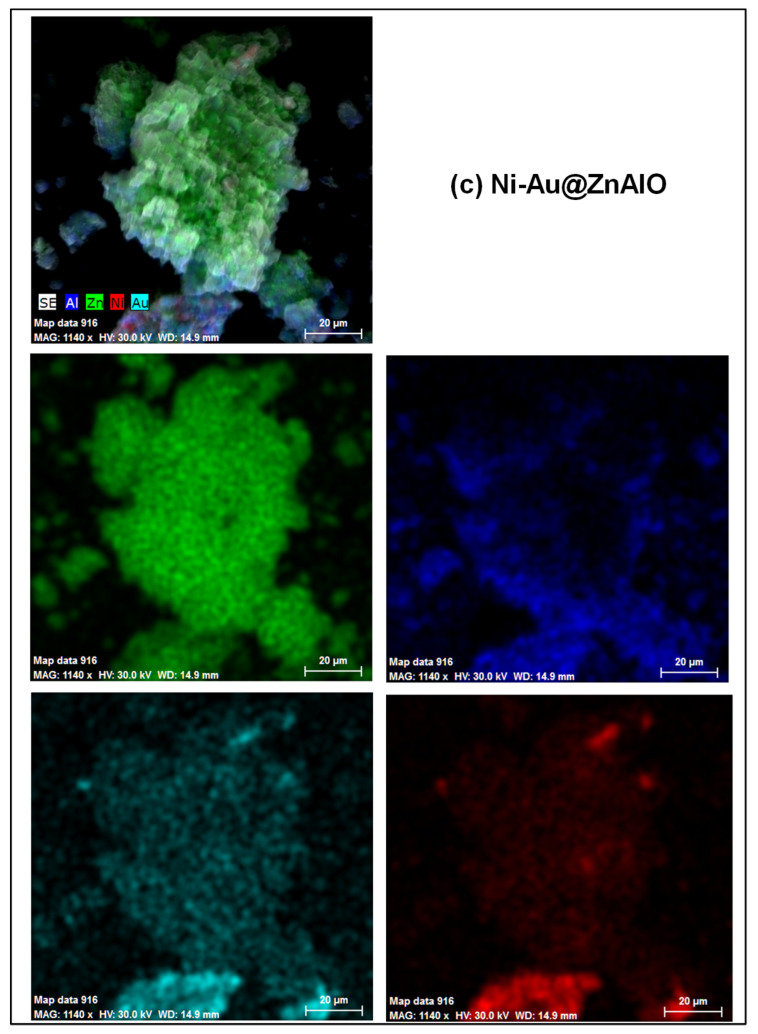
Elemental mapping images for (**a**) Ni@ZnAlO, (**b**) Au@ZnAlO, and (**c**) Ni-Au@ZnAlO catalysts.

Elemental mapping by energy-dispersive X-ray spectroscopy (EDS) for the mono- and bimetallic-based catalysts (Figure 3a–c) reveals the distribution of Zn, Al, Ni, and Au elements throughout the Ni@ZnAlO, Au@ZnAlO, and Ni-Au@ZnAlO samples, and therefore, the successful synthesis of the nanocomposite materials.

Given that a small amount of metals was used for the synthesis of nanoparticles, the XRD pattern could not identify the diffraction lines of Ni^0^ in the Ni-Au@ZnAlO solid. However, the EDAX observations agree with the XPS measurements confirming the presence of mono- and bimetals in the targeted samples, as will be shown in the following.

### 2.3. Infrared Spectroscopy

**Figure 4:**
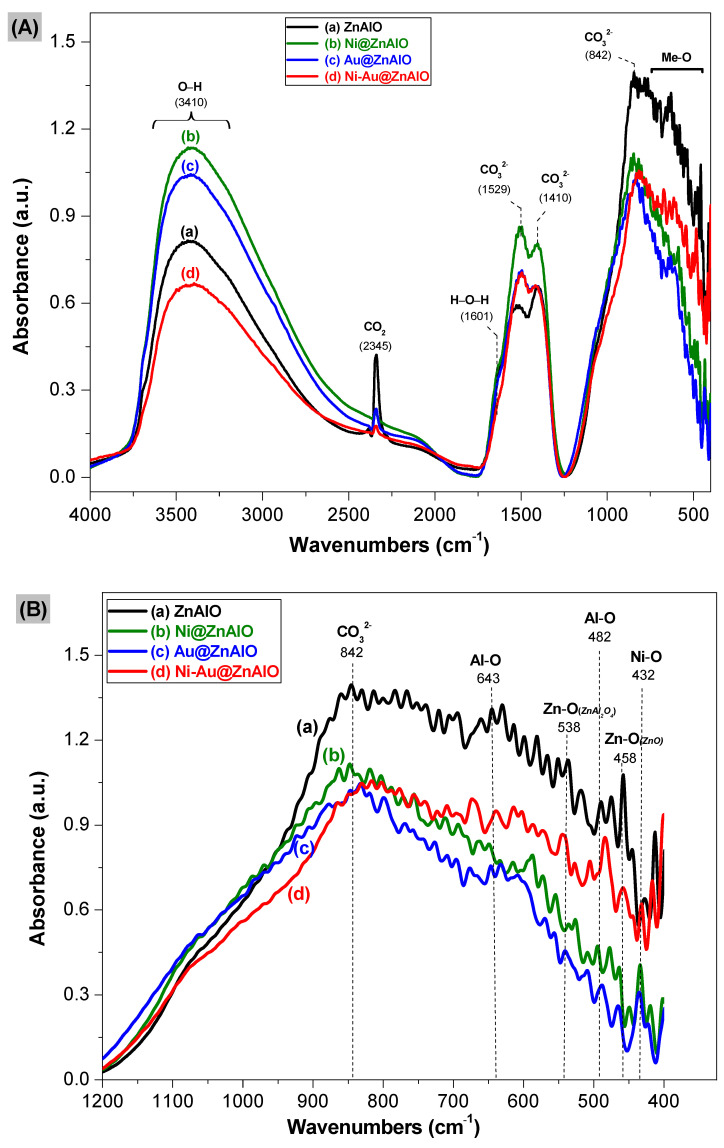
The DRIFT spectra of the obtained catalysts (**A**) for the full spectra and (**B**) DRIFT region from 1200 cm^−1^–400 cm^−1^.

The synthesized nanocomposites exhibited morphological features correlated directly to their structural and optical properties. The DRIFT (Diffuse Reflectance Infrared Fourier Transform) spectra of free ZnAlO and those of NPs-containing ZnAlO catalysts recorded in the region 400–4000 cm^−1^ are displayed in Figure 4A, while the region below 1200 cm^−1^ is shown in Figure 4B. The highest frequency region of all samples reveals a broad band with a maximum at 3410 cm^−1^ assigned to the stretching vibration of OH groups (O-H). The band centered at 1601 cm^−1^ is responsible for the bending mode of water molecules (H-O-H). Notably, the vibration band of the –OH group (e.g., 3410 cm^−1^) increased in intensity when the monometallic nanoparticles were added over the ZnAlO support, whereas this band decreased after the Ni-Au bimetallic NPs loading. The vibration bands appearing at 1529 cm^−1^ and 1410 cm^−1^ are assigned to the bending and stretching vibrations of CO_3_^2−^ species arising from the initial LDH structure of the support, as confirmed by the XRD analysis. The sharp band observed at 2345 cm^−1^ for the ZnAlO matrix relates to the asymmetric stretch of CO_2_ adsorbed on the catalyst’s surface [33,34]. The two bands in the region 1750–1250 cm^−1^ increased in intensity after the addition of metallic nanoparticles compared to the ZnAlO solids. Meanwhile, the case of the band at 2345 cm^−1^ varies inversely. Looking at the lower wavenumbers, the position at 842 cm^−1^ corresponds to the out-of-plane bending vibrations of the carbonate ions. The band appearing at 643 cm^−1^ indicates the Al-O stretching vibrations with octahedral coordination (AlO_6_), and the band positioned at 482 cm^−1^ corresponds to bending Al-**O** bonds with octahedral coordination [35], while the vibration at 538 cm^−1^ is attributed to Zn-O, indicating the characteristic vibration bands in ZnAl_2_O_4_ spinel structure. For the wavenumber of 432 cm^−1^, the characteristic band of Ni-O is evidenced [36], whereas Zn-O vibrational band from ZnO phase was identified at 458 cm^−1^ [37]. Note that the characteristic band of Zn-O is prominent for ZnAlO support material and decreases its intensity after the loading of the metallic nanoparticles. According to Davar and Niasari [38], the ZnAl_2_O_4_ spinel structure revealed characteristic vibrations at 490, 540, and 652 cm^−1^ associated with the Al-O bending vibrations, Zn-O stretching vibrations, and Al-O stretching vibrations. For both ZnAl_2_O_4_ and ZnO structures, the Zn^2+^ cations occupy the tetrahedral sites of each corresponding lattice; however, their Zn-O bond has different lengths. The bands related to the ZnAl_2_O_4_ phase found in this study match with those from the literature data. Moreover, the Zn-O vibrational band from the ZnO phase (458 cm^−1^) is shifted to higher wavenumbers in the ZnAl_2_O_4_ structure (538 cm^−1^) due to the change in Zn-O bond length by the presence of aluminum. These observations are correlated with the UV-Vis spectra of the materials, confirming the existence of Zn^2+^ in two different structures.

In this study, it could not be distinguished for the Au-O bond because more valuable information regarding the identification of Au species was obtained using the IR spectroscopy of adsorbed CO molecule [39]. However, the obtained results from complementary techniques (e.g., EDX, XRD, TPR, UV-Vis) certify the existence of gold in the targeted catalysts. Additionally, the XPS analysis matches the DRIFTs data, confirming the successful formation of NPs@ZnAlO nanocomposites.

The DRIFTs measurements were further corroborated with the ATR (Attenuated Total Reflectance) spectra (Figure S1). The main observed ATR band with a maximum at 3519 cm^−1^ is attributed to O-H bonds characteristic of surface OH groups, while the absorption band at 1518 cm^−1^ corresponds to the antisymmetric stretching vibrations of CO_3_^2−^ intercalated between the layers of the ZnAl mixed oxide. The two bands observed at 2315 and 2368 cm^−1^ are associated with the stretching vibrations of weakly adsorbed CO_2_ on the surface of catalysts. These bands increased after the mono- and bimetals were loaded on the ZnAlO support.

### 2.4. UV-Vis Spectroscopy

**Figure 5:**
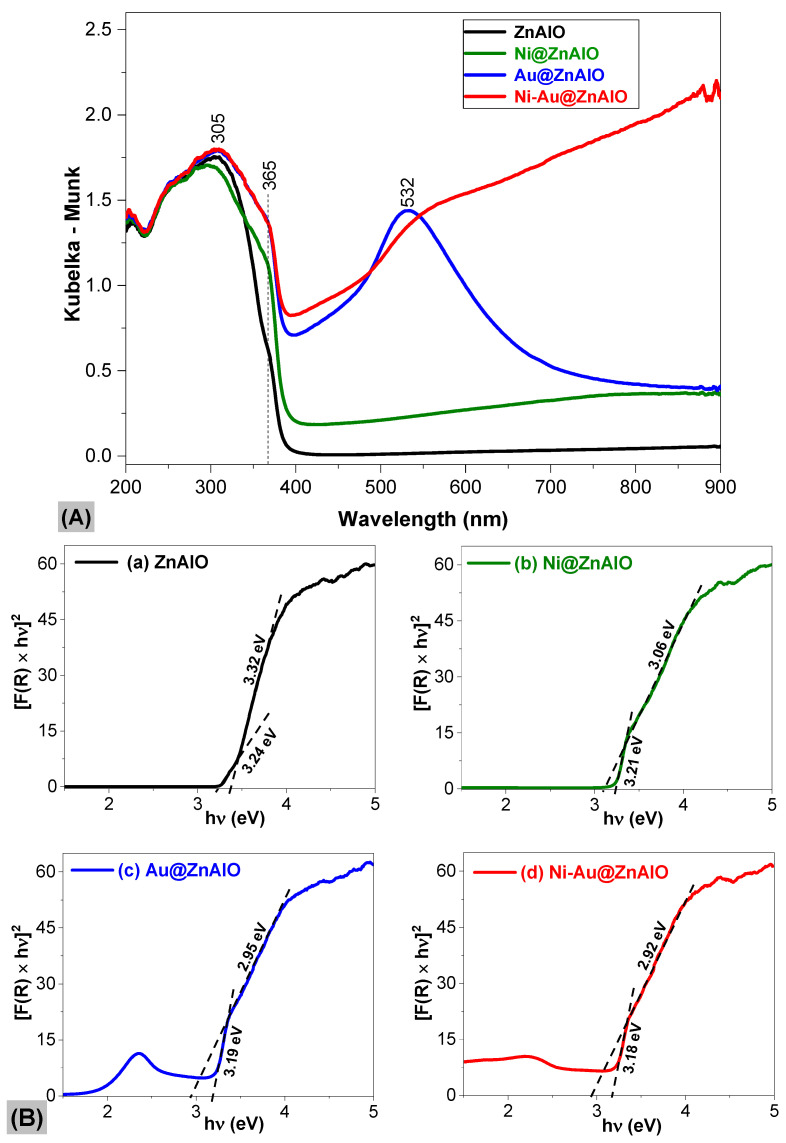
The UV-Vis absorption spectra of: (**A**) ZnAlO, Ni@ZnAlO, Au@ZnAlO, and Ni-Au@ZnAlO photocatalysts; and the (**B**) Tauc plot of (**a**) ZnAlO, (**b**)Ni@AZnAlO, (**c**) Au@ZnAlO, and (**d**) Ni-Au@ZnAlO, representing the two edges of the samples considered for their band gap estimations.

The optical features of free ZnAlO and those of NPs-containing ZnAlO mixed oxides are displayed in Figure 5A, and the band gap values estimated by the Tauc plot [40] are shown in Figure 5B. According to the UV-Vis absorption spectra, all the prepared samples exhibit absorption edges around 305 and 365 nm associated with electron transfer from the valence band to the conduction band (O2p→Zn3d) [41]. After loading the metallic NPs over the ZnAlO matrix, an improvement in the light harvested was observed. Additionally, the spectra of Au@ZnAlO revealed a shoulder at 532 nm assigned to the surface plasmonic resonance (SPR) excitation, characteristic of gold NPs. The SPR band was slightly shifted in the case of the Ni-Au@ZnAlO sample, due to both the bimetallic nanoparticle formation and their sizes. The work of Barnawi et al. [42] admitted that the SPR band of Au NPs moved to a higher wavelength (red-shift) for samples with larger particle sizes, while a blue-shifted position was noticed in the samples with smaller particle sizes. Comparatively, the UV-Vis spectra of Ni NPs, Au NPs, Ni-Au NPs, and ZnAl-LDH samples are given in Figure S2.

For the free ZnAlO and those of NPs-containing ZnAlO materials, the presence of two edges is observed: the large slope is due to ZnO, whereas the small slope is given by the ZnAl_2_O_4_ spinel phase. Indeed, upon calcination of LDH precursors at temperatures higher than 500 °C, Al^3+^ ions migrate from the initial ZnO-like structure to form the ZnAl_2_O_4_ spinel phase [43]. Given that the diffraction lines of ZnAl_2_O_4_ are very close to some of those of ZnO [44], it is reasonable to assume that small amounts of ZnAl_2_O_4_ might be uniformly distributed on the surface of NPs@ZnAlO and/or exist as an amorphous phase that is not detectable by XRD analysis.

The reported literature for the band gap value of ZnAl_2_O_4_ in bulk form is around 3.8 eV [45], and the band gap of ZnO is ≈3.4 eV [46], whereas mixing of multiple oxides will further decrease the band gap. The obtained values attributed to ZnO followed the trend of Ni-Au@ZnAlO (2.92 eV) < Au@ZnAlO (2.95 eV) < Ni@ZnAlO (3.06 eV) < ZnAlO (3.32 eV). These energy values are close to those reported by Zhang et al. [47], which are similar to the band gap of M/Cu/ZnO (M = Ag, Ni, Fe) photocomposites [48]. The Eg calculated for the ZnAl_2_O_4_ changed from Ni-Au@ZnAlO (3.18 eV) ≈ Au@ZnAlO (3.19 eV) < Ni@ZnAlO (3.21 eV) < ZnAlO (3.24 eV). Similar behavior was reported by Lopez et al. [49] from ZnO-ZrO_2_ nanocomposites used for the photodegradation of phenol. In this study, the deposition of nanoparticles over mixed oxides-derived LDH precursors led to the improvement in both the optical properties and photo-carrier collection, enhancing the performance of the photocatalysts.

### 2.5. X-Ray Photoelectron Spectroscopy (XPS) Studies

**Figure 6:**
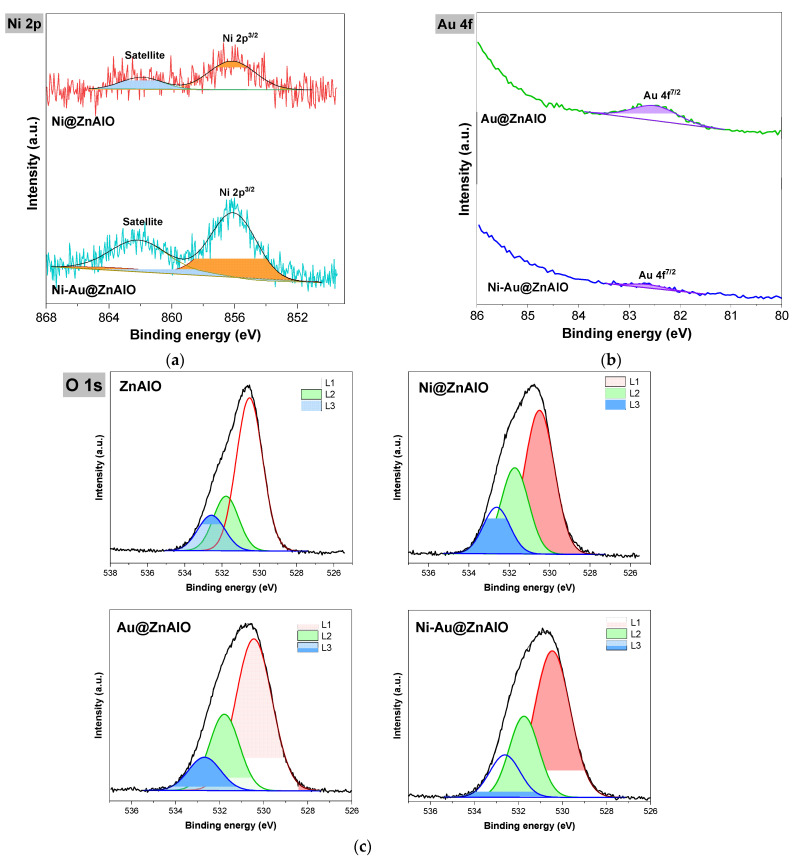
XPS analysis of catalysts showing the spectra of (**a**) Ni 2p for Ni@ZnAlO and Ni-Au@ZnAlO; (**b**) Au 4f for Au@ZnAlO and Ni-Au@ZnAlO; and (**c**) O 1s for ZnAlO, Ni@ZnAlO, Au@ZnAlO, and Ni-Au@ZnAlO materials.

XPS analysis was employed to examine the surface composition and possible surface metal interactions. The deconvolution of the XPS spectrum of Ni 2p for both Ni@ZnAlO and Ni-Au@ZnAlO (Figure 6a) exhibits one peak at 856.1 eV corresponding to Ni 2p^3/2^, while at 861.9 eV, the satellite peak of nickel oxide is observed. The Au 4f spectrum of Au@ZnAlO and Ni-Au@ZnAlO solids (Figure 6b) showed one peak at 82.5 eV, which is assigned to Au 4f^7/2^. The intensity of this peak is lower in the case of the bimetallic-based catalyst compared to the monometallic counterpart, and this behavior could be due to the covering of surface nickel nanoparticles by Au NPs.

The high-resolution XPS spectrum of O 1s of ZnAlO, Ni@ZnAlO, Au@ZnAlO, and Ni-Au@ZnAlO (Figure 6c) was deconvoluted into three peaks at 530.5, 531.7, and 532.5 eV, respectively. The first peak (denoted as L1) corresponds to the lattice oxygen [50]; the second peak (labeled as L2) is attributed to water molecules strongly bound to the exposed ZnO surface [51]; and the third peak (marked as L3) is associated with the existing loosely bound surface oxygen (adsorbed OH/H_2_O) [52].

XPS of Zn 2p (Figure S3) for all the samples is found at 1022 eV, confirming the existence of the Zn^2+^ oxidation state from the ZnO [53]. The core-level spectrum of Zn LMM Auger was performed to identify defects like interstitial zinc (Zn_i_) or zinc bonded with oxygen. Analysis of all the samples led to the deconvolution of Zn LMM Auger into five peaks. Typical PA (parameter Auger) values (see Table S1) for ZnO are in the range of 2010–2010.5 eV, whereas for ZnCO_3,_ they are even lower. Higher values, as obtained in the case of the present study, can be attributed to the presence of Al in the structure. This decrease in the PA from ZnAlO and Ni@ZnAlO samples to the Au@ZnAlO and Ni-Au@ZnAlO solids may be caused by some amount of carbonate on the surface. The DRIFT spectra confirm the presence of carbonate in the samples.

The Al 2p core-level spectra obtained by XPS for the obtained samples (Figure S4) reveal one peak at around 73.8 eV, which corresponds to the Al^3+^ oxidation state of the samples. The measured binding energy (e.g., 73.8 eV) is slightly lower than that of Al_2_O_3_ (74 eV), confirming that it did not form a distinct alumina phase. Jain et al. [45] admitted that this peak originates from the octahedral position of Al in the ZnAl_2_O_4_ lattice. Indeed, this observation matches the UV-Vis results, validating that all catalysts displayed a large slope attributed to the ZnO, while the small slope was due to the ZnAl_2_O_4_ phase.

### 2.6. H_2_-TPR Measurements

**Figure 7:**
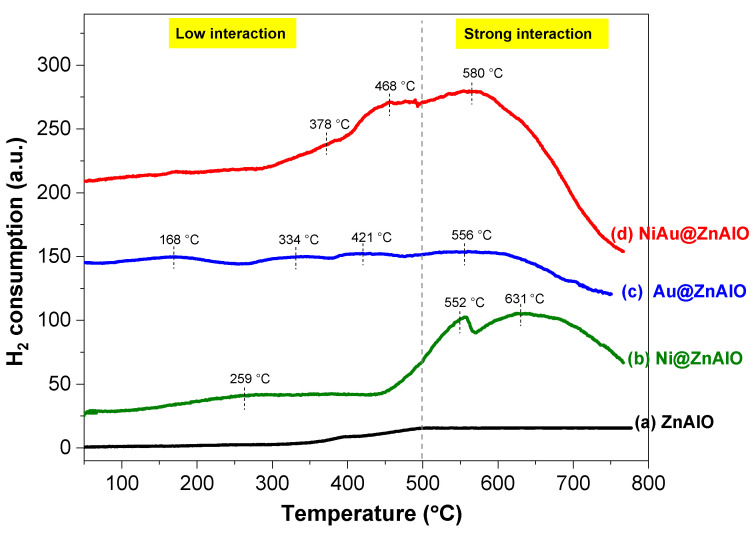
The H_2_-TPR profiles of the ZnAlO matrix and the NPs-modified catalysts.

H_2_-TPR (temperature programmed reduction) measurements were used to examine the reducibility behavior of mono- and bimetallic NPs in the ZnAlO mixed oxide catalysts, as well as their metal–support interaction (Figure 7). The NPs-free sample (e.g., ZnAlO) confirmed that no species can be reduced over this solid in the studied temperature range. The TPR profiles of the NPs containing catalysts show different reduction behaviors related to the type of added nanoparticles (mono-, or bimetallic NPs). The profile of the Au@ZnAlO sample reveals three reduction maxima. The low-temperature reduction peak, occurring at approximately 168 °C, is attributed to the reduction in Au^3+^ to Au^+^. The second hydrogen consumption peak, which appears at 334 °C, corresponds to the reduction in Au^+^ to Au^0^ [54]. The high-temperature peaks at 421 and 556 °C can be attributed to the reduction of oxygen in the ZnO lattice, which is favored by the presence of gold on the surface [55]. After the deposition of nickel on the mixed oxides support (e.g., Ni@ZnAlO), three peaks are observed. The reduction in NiO surface species is attributed to the two reduction peaks at low temperatures, which are centered at 259 °C and 552 °C. This reduction can be associated with either isolated species NiO or NiO that is unevenly dispersed on the weakly interacting ZnAlO surface [56]. The third hydrogen consumption located at 631 °C relates to the strong interaction of Ni^2+^/Ni^0^ species with the support.

Research studies indicate that Ni@ZnO systems demonstrate strong metal–support interactions (SMSI), which significantly influence their catalytic performance [57]. The bimetallic-containing catalyst displays a similar reduction behavior to that of the monometallic Ni@ZnAlO material. Therefore, the shoulder positioned at 378 °C in the Ni-Au@ZnAlO catalyst is assigned to Au^+^ species. The reduction peak at about 468 °C is due to Ni^2+^/Ni^0^, and the peak at the highest temperature (maximum at 580 °C) corresponds to the strong metal–support interaction. Furthermore, as previously demonstrated in the literature [58], the addition of a second metal (such as Au) to the ZnAlO mixed oxide demonstrates that the existence of gold promotes the nickel reduction.

Table 2 presents experimental quantitative results obtained through selective hydrogen chemisorption and programmed thermal reduction (H_2_-TPR). The hydrogen chemisorption provides insights into the dispersion of nickel on the mixed oxide ZnAlO. It is observed that the dispersion of nickel is better in the catalyst containing bimetallic nanoparticles. Additionally, the surface area of exposed metallic nickel is larger for the bimetallic catalyst Ni-Au@ZnAlO compared to Ni@ZnAlO. The XPS results confirm an increase in nickel concentration on the surface of the Ni-Au@ZnAlO catalyst, which is correlated with the XRD RIR method (see Table 1). This can be explained by the Ostwald ripening phenomenon, wherein small nickel particles from the colloidal solution migrate and deposit onto larger particles, enveloping the gold nanoparticles that have increased in size [59].

It should be noted that the H_2_ chemisorption results are reported only for the Ni@ZnAlO solid because the larger supported gold nanoparticles do not directly absorb hydrogen molecules under the identical experimental conditions [60].

### 2.7. Photocatalytic Degradation of Bisphenol A (BPA) Under Simulated Solar Irradiation

**Figure 8:**
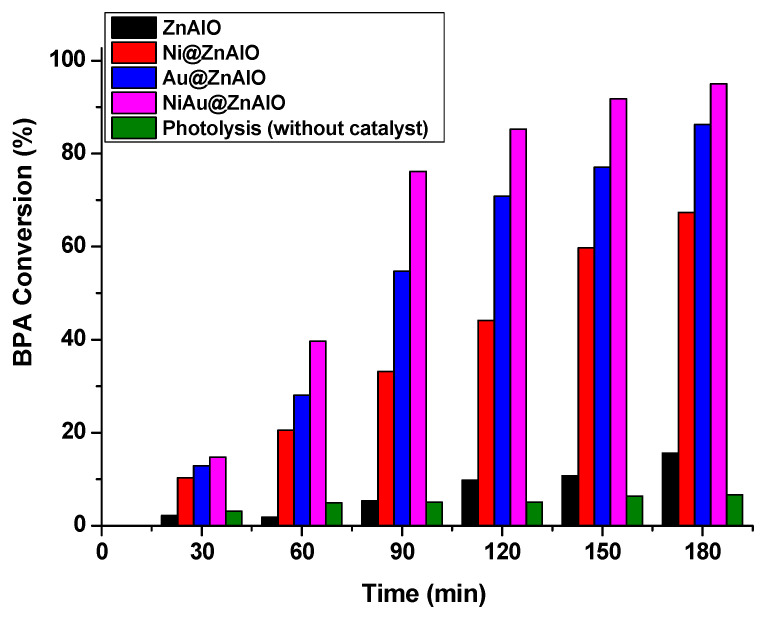
Time-dependent conversion of BPA (in percentage).

**Figure 9:**
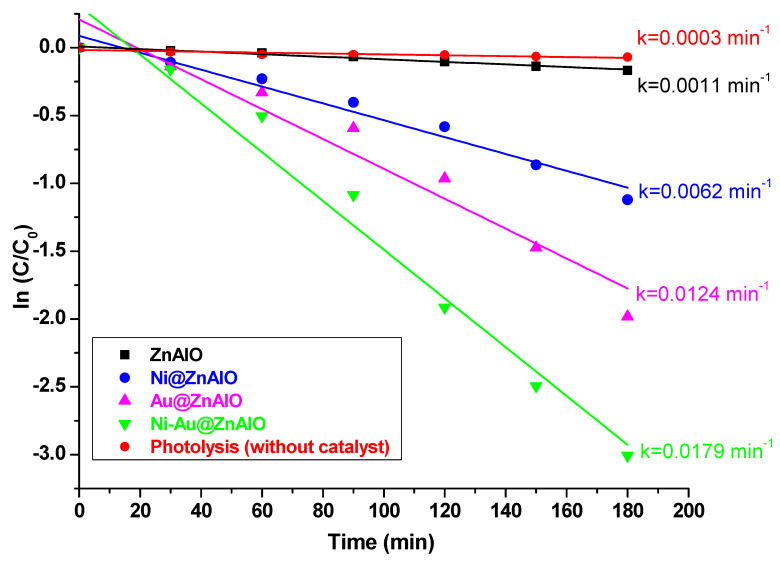
ln (C/C_0_) versus irradiation time.

The photocatalytic performance was evaluated for the NPs-free ZnAlO and NPs-containing ZnAlO catalysts, as well as in the absence of a catalyst, following the degradation of bisphenol A (BPA) pollutant under simulated solar light (SSL) irradiation. The time-dependent conversion of BPA over ZnAlO, Ni@ZnAlO, Au@ZnAlO, Ni-Au@ZnAlO, and photolysis (without catalyst) is given in Figure 8. The photocatalytic activity order towards BPA degradation evolved as follows: Ni-Au@ZnAlO > Au@ZnAlO > Ni@ZnAlO > ZnAlO conversion of 95.05%; 86.22%; 67,38%; 15.67%; and 6.64% after 180 min of irradiation. Photoexcited electrons were generated from surface plasmon-active Au nanoparticles and the semiconductor ZnAlO. The synergistic effect between Au and Ni enhanced charge separation. The Ni-Au@ZnAlO catalyst was able to degrade 95% of the BPA pollutant during the exposed period, compared to the ZnAlO matrix (15.6%) (Table 3). Since the bimetallic nanoparticles can act to trap photo-induced electrons, the recombination of electron–hole pairs is slowed, thereby improving the photoactivity. The photocatalytic performance of the studied catalysts was further evaluated by determining the reaction rate constant (k) for the photodegradation of BPA. This was assessed using pseudo-first-order kinetic models [61]. Figure 9 depicts the correlation between ln(C/C_0_) and time, highlighting the linear fitting curves for photocatalytic degradation across NPs@ZnAlO catalysts and non-catalytic processes (photolysis).

The apparent calculated kinetic rate constant (k) for Ni-Au@ZnAlO was 0.0179 min^−1^, markedly higher than for ZnAlO, which was only 0.0011 min^−1^. This behavior is due to improved light absorption, narrower band gap, and a lower recombination rate of photo-induced electron–hole (e^−^/h^+^) pairs (better charge separation). Ni and Au NPs can extend the light absorption of ZnAlO into the visible range due to surface plasmon resonance (SPR), especially from Au. This enables the photocatalyst to absorb a wider range of the solar spectrum, thereby increasing photoexcitation and, consequently, the reaction rates. The degradation efficiency of monometallic NPs containing photocatalysts reached 0.0062 min^−1^ and 0.0124 min^−1^ for Ni@ZnAlO and Au@ZnAlO, respectively.

Catalytic tests were conducted in the absence of light under the same experimental conditions for both the Ni-Au@ZnAlO catalyst and the ZnAlO support (see Figure S5). The results indicate that BPA is not converted in the absence of simulated solar light with degradation conversions remaining below 1%. These findings confirm that light irradiation plays a crucial role in activating the catalysts.

**Figure 10:**
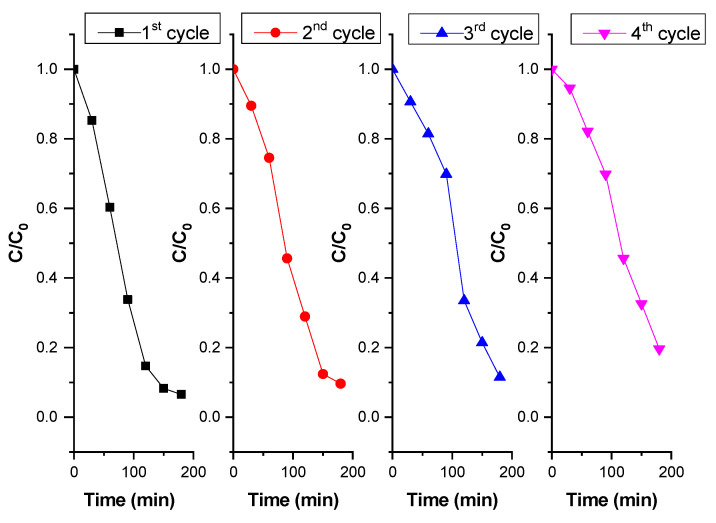
Stability studies on the Ni-Au@ZnAlO photocatalyst for the photodegradation of BPA.

Photocatalyst stability is critical for maintaining and increasing the effectiveness of photocatalytic processes. Additional catalytic studies were performed to assess the stability of the Ni-Au@ZnAlO sample, which was selected for its high photocatalytic activity for BPA degradation. The four experiments were performed using a catalyst dosage of 0.05 g in 110 mL and 25 mg L^−1^ BPA concentration. The catalyst was recovered after each test reaction, cleaned with distilled water, topped at a dosage of 0.454 g L^−1^, and reused in the reaction mixture. The results, after four reuse tests, are reported in Figure 10 and Figure S5 in terms of C/C_0_ and % conversion of BPA removal versus time, respectively.

The BPA degradation conversion varies from 95.05% (first cycle) > 93.51% (second cycle) > 91.8% (third cycle) > 91.2% (fourth cycle), indicating a slight decrease. According to an examination of the experimental data, the Ni-Au@ZnAlO catalyst demonstrated good stability, which makes it a promising candidate for a wide range of catalytic and photocatalytic applications.

The intermediate compounds resulting from the photodegradation of Bisphenol A (BPA) over the obtained NP@ZnAlO nanocomposites were identified in this study using HPLC and GC-MS techniques. The identified compounds include 4-isopropenyl phenol, 4-isopropropyl phenol, phenol, dihydroxylated bisphenol, dihydroxybenzene, hydroquinone, and various aliphatic acids. Similar results were reported by Velumani et al. [62], who identified four main intermediates during the photocatalytic degradation of BPA on the c-SB/ZnO composite, including 4-isopropenyl phenol, 4-isopropyl phenol, benzophenone, and dihydroxylated bisphenol. According to the results obtained by the research group mentioned above, BPA degradation pathways occur via ring cleavage/opening reactions, mineralization, hydroxylation, and oxidation steps. The first step involves the addition of OH^−^ radicals to the aromatic rings of BPA, leading to the formation of mono-, and dihydroxylated BPA. The second pathway refers to the formation of the 4-isopropenylphenol/4-isopropylphenol and phenol from the BPA molecule as a result of the OH^−^ attack on the phenyl group, which further converts to benzophenone. In the last step, aliphatic compounds (e.g., formic acid) will form an oxidative ring-opening reaction structure, followed by mineralization to harmless end products (e.g., CO_2_, H_2_O). A similar mechanism was reported by Mahmoudian and co-workers [63], confirming that the photodegradation of BPA is achieved through hydroxyl radicals (OH^−^) and holes (h^+^), acting as powerful oxidizing species in the advanced photocatalytic processes [64,65].

**Scheme 1:**
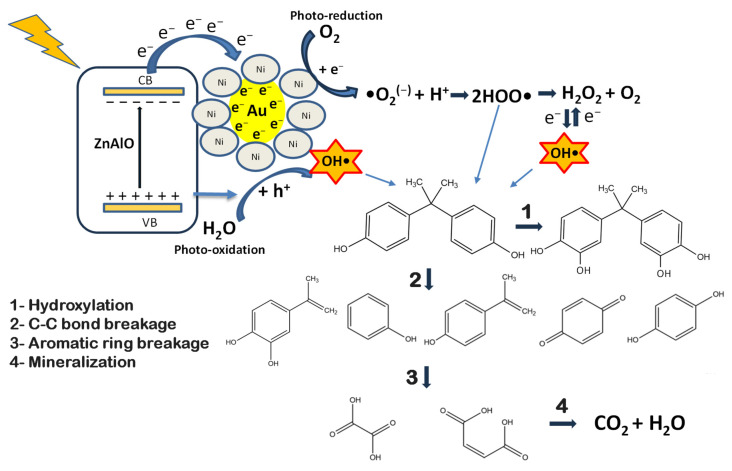
Proposed reaction mechanism for the photodegradation of BPA over Ni-Au@ZnAlO photocatalyst.

Based on the obtained results, we propose a modified reaction mechanism for the degradation of Bisphenol A over the NPs@ZnAlO nanocomposites, as illustrated in Scheme 1.

When sunlight irradiates the ZnAlO catalyst, it generates photogenerated electrons (e^−^) and holes (h^+^), which migrate to the surface and create a strong redox potential. This process allows the ZnAlO photocatalyst to facilitate redox reactions by producing electron–hole pairs. When photons with sufficient energy interact with the photocatalyst, the electrons are excited from the valence band (VB) to the conduction band (CB). The holes left in the VB then oxidize donor molecules, while the electrons in the CB reduce oxygen, resulting in the formation of superoxide ions. These electron–hole pairs initiate photoreduction and photo-oxidation reactions, leading to the degradation of contaminants and their demineralization. The photoexcited electrons on the Ni-Au@ZnAlO catalyst are generated by surface plasmon activity of gold nanoparticles and the ZnAlO semiconductor. The synergistic effect between Au and Ni enhances the charge separation, leading to improved catalytic efficiency for BPA degradation.

The photocatalytic degradation of bisphenol A (BPA) on Ni-Au@ZnAlO (Scheme 1) follows a multi-step oxidative pathway: In the initial stage, hydroxyl radicals (OH^−^) attach to the aromatic rings of BPA, leading to the formation of mono- and dihydroxylated BPA derivatives. The next step involves the attack of OH^−^ radicals on the phenyl groups of BPA, resulting in the formation of intermediates such as 4-isopropenylphenol, 4-isopropylphenol, phenol, hydroquinone, and dihydroxibenzene. In the final stage, oxidative ring-opening reactions generate aliphatic by-products, such as oxalic and maleic acid. These compounds are further mineralized into environmentally benign end products, carbon dioxide (CO_2_) and water (H_2_O).

In the Ni-Au@ZnAlO bimetallic catalyst, nickel facilitates electron transfer to gold (Au), owing to their differing redox potentials. This transfer elevates the electron density on the gold surface, modifying the Fermi level and promoting enhanced electron donation to adsorbed molecules [66]. Consequently, this synergistic interaction boosts the catalyst’s effectiveness in degrading bisphenol A (BPA).

Comparative studies of the current and previous literature data regarding the photodegradation of bisphenol A over several photocatalysts are given in Table 4.

Therefore, well-dispersed nanoparticles loaded over homogeneous mixed oxides provide robust materials with a modulated band structure capable of degrading pollutants.

## 3. Materials and Methods

### 3.1. Synthesis of Materials

**Step (i): Synthesis of mono-(Ni, Au) and bimetallic (Ni-Au) nanoparticles (NPs) by the polyol alkaline method**. In a typical procedure, monometallic and bimetallic nanoparticles protected with polyvinylpyrrolidone (PVP) (M.W. 8000, Alfa-Aesar, Kandel, Germany) were prepared following a modified version of the alkaline polyol method [21,72]. Ethylene glycol (Lachner-Neratoviche, Czech Republic) served as both the solvent and reducing agent. For this synthesis, the solutions were prepared in advance: a nickel (Ni^2+^) solution at a concentration of 10^−2^ M in ethylene glycol (EG) from Ni(NO_3_)_2_·6H_2_O Sigma-Aldrich, Darmstadt, Germany precursor and a gold (Au^3+^) solution at a concentration of 10^−2^ M in EG from Au^3+^ (HAuCl_4_ Sigma-Aldrich, Taufkirchen, Germany. An alkaline solution of polyvinyl pyrrolidone (PVP) in EG was also prepared by dissolving polyvinylpyrrolidone at 50 °C. Sodium hydroxide was then added to create a solution with a concentration of 0.1 M PVP and 0.1 M NaOH (Merck, Darmstadt, Germany) in ethylene glycol. The experimental procedure for synthesizing monometallic nanoparticles is as follows: A 10 mL solution containing Ni^2+^ or Au^3+^ precursor, dissolved in ethylene glycol at a concentration of 10^−2^ M, is mixed at room temperature with a 10 mL alkaline NaOH solution (0.1 M PVP; 0.1 M NaOH under H_2_/Ar atmosphere). After deaeration, the solution is rapidly heated up to 120 °C under stirring. The initially transparent solution changes to gray for nickel and to ruby-red for gold at approximately 70 °C, indicating the reduction in the metal. Nucleation and growth of Ni (or Au) particles occur at 140 °C for 120 min.

For bimetallic nanoparticles, 10 mL of Ni^2+^/EG, 10^−2^ M and 10mL Au^3+^/EG, 10^−2^ M (1:1 molar ratio) is mixed with an alkaline PVP/EG solution (0.1 M PVP and 0.1 M NaOH) at a molar ratio of PVP to metal (M) of 10. This mixture is heated in a reducing atmosphere to 140 °C for 120 min to facilitate nanoparticle maturation. The colloidal solutions of both monometallic and bimetallic nanoparticles are then cooled in a 5% H_2_/Ar atmosphere, mixed with acetone, and left at −16 °C for 24 h. After separation by centrifugation, the particles are washed several times with acetone to remove excess ethylene glycol and PVP, and then dried for 8 h at 100 °C.

**Step (ii): Synthesis of ZnAl LDH precursor and its corresponding mixed oxide**. The ZnAl layered double hydroxide (LDH) was prepared through the coprecipitation method at constant pH (9.5) using Zn(NO_3_)_2_·6H_2_O and Al(NO_3_)_3_·9H_2_O as metal precursors (solution A). The aqueous precipitating solution consisted of 1 M NaOH and 0.2 M Na_2_CO_3_ (solution B). The molar ratio of M^2+^/M^3+^ was kept at 3. Both solutions were simultaneously added dropwise into a beaker and mixed at room temperature under vigorous stirring. The obtained slurry was placed into a three-neck flask and aged at 75 °C for 24 h, maintaining the stirring. The suspension was then centrifuged, washed with deionized water until a neutral pH, and finally dried at 110 °C overnight in an air atmosphere. This solid is identified as ZnAl LDH. The calcination of the LDH precursor at 650 °C for 6 h in an air flow led to its corresponding mixed oxide denominated as ZnAlO.

**Scheme 2:**
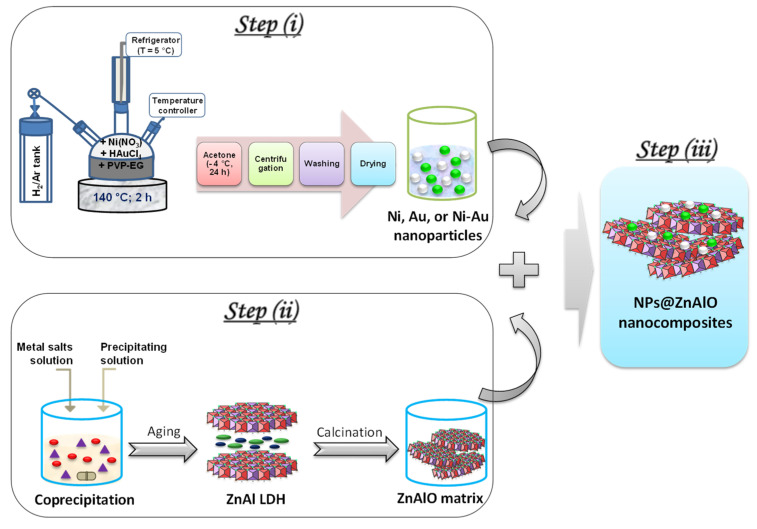
Synthesis steps for the fabrication of NPs@ZnAlO nanocomposite materials.

**Step (iii): Controlled assembling of NPs@ZnAlO nanocomposites.** In this step, the mono-(Ni, Au), and bimetallic (Ni-Au) nanoparticles were assembled in a controlled manner within the ZnAlO support to achieve NPs@ZnAlO nanocomposites. The NPs were then loaded onto ZnAlO mixed oxide supports and calcined at 350 °C for 2 h under air flow to remove the residual PVP, resulting in a total metal loading of 1wt.% Ni@ZnAlO, 1wt.% Au@ZnAlO, and 1wt.% (Ni+Au)@ZnAlO. These materials are identified as Ni@ZnAlO, Au@ZnAlO, and Ni-Au@ZnAlO. The complete fabrication process of the NPs@ZnAlO nanocomposite materials is illustrated in Scheme 2.

### 3.2. Characterization of the Photocatalysts

The powder X-ray diffractograms (XRD) were acquired using a Rigaku Corporation Ultima IV diffractometer (Tokyo, Japan) with monochromatic Cu Kα radiation. The crystallite size was estimated using Williamson-Hall’s equation. The microstructure of the obtained samples was analyzed using a Tescan Vega 3LMH scanning electron microscope coupled with EDAX (Brno, Czech Republic). UV-Vis spectra were acquired on a Perkin Elmer Lambda 35 spectrophotometer (Shelton, CT, USA) equipped with an integrating sphere. Tauc plots were plotted as [F(R)·hν]^2^ versus hν (eV) for direct band gap transitions from the inflection point tangent to the linear portion of the absorption curve. The diffuse-reflectance infrared (DRIFT) spectra were obtained with JASCO FT/IR-4700 spectrometer (Jasco, Tokyo, Japan) by the acquisition of 128 scans in the 4000–400 cm^–1^ wavelength domain. Attenuated total reflectance Fourier transformed infrared (ATR-FTIR) spectra were measured with a JASCO FT/IR-4700 spectrometer equipped with a diamond crystal using a scanning speed of 128 scans·min^–1^, triangle apodization, and a resolution of 4 cm^−1^. Textural properties were measured by N_2_ physisorption isotherms at liquid nitrogen temperature using a Micromeritics ASAP 2010 apparatus., Norcross GA, USA Before analysis, the samples were degassed under vacuum at 200 °C for 2 h. X-ray photoelectron spectroscopy (XPS) measurements were obtained on a SPECS spectrometer (Berlin, Germany) with a PHOIBOS 150 analyzer (Berlin, Germany). The acquisitions of data were operated at a pass energy of 20 eV for the individual spectral lines and 50 eV for the extended spectra. A monochromatic radiation source of Specs XR-50M type (E_x_ = 1486.7 eV) operating at 250 W was employed. The charge compensation was completed by a flood gun of Specs FG15/40 type. The C 1s peak at 284.6 eV was used as a reference for all recorded spectra. In the XPS analysis, the Zn LMM Auger electron spectra (AES) was also considered for the study of the chemical state of zinc, while the modified Auger parameter was calculated according to Equation (1):

PA = BE (Zn2p^3/2^) + KE(max (Auger Zn LMM)) (1)

The temperature-programmed reduction (H_2_–TPR) measurements were carried out with a CHEMBET-3000 Quantachrome Instrument (Boynton Beach, FL, USA) furnished with a thermal conductivity detector (TCD). In every experiment, 50 mg of the sample was placed in the quartz reactor and heated up to 800 °C (rate of 10 °C·min^−1^) using a 5% H_2_/Ar gas flow (90 mL·min^−1^). The H_2_ consumption was estimated from the area of the recorded peaks. The experimentally obtained peak surface (mV∙s) was converted into micromoles of hydrogen. Hydrogen chemisorption was conducted using the same equipment. The nickel-containing samples were reduced at 400 °C before chemisorption and then kept in an inert helium flow to eliminate any physically adsorbed hydrogen species. After establishing a baseline, hydrogen pulses were injected to facilitate the dissociative adsorption of hydrogen at 25 °C. By measuring the amount of adsorbed hydrogen, we can calculate the exposed metal surface area and the nickel dispersion on the surface.

### 3.3. Photocatalytic Experiments

The photocatalytic degradation of Bisphenol A (BPA) under simulated solar irradiation was performed in a quartz photoreactor thermostated at 20 °C. The light source was generated by a solar simulator (AM 1.5 Peccell-L01, Yokohama, Japan) equipped with a 150 W Xe short-arc lamp (1000 W·m^−2^). In a typical test, 0.050 g of the photocatalyst are dispersed in 110 mL of aqueous BPA solution (25 mg·L^−1^, pH = 6.7) while maintaining continuous stirring. The photoreactor is provided with a quartz window of 4.5 × 4.5 cm^2^ for light irradiation. Before irradiation, the samples were kept in the dark for 40 min, under stirring, to reach the absorption/desorption equilibrium. At each half hour, an appropriate amount of the sample was taken and filtered for subsequent analysis.

The evolution of the liquid phase (BPA and the reaction products) was monitored by a high-performance liquid chromatograph (HPLC) (Waters, Alliance e2659, Milford, MA, USA) equipped with UV-Vis detector (λ = 275 nm) (Waters, model 2489), column Kromasil 100 5–C18, mobile phase acetonitrile/water = 60:40 (*v*/*v*), flow rate 1 mL·min^−1^, column temperature 35 °C, and an injection volume of 2 µL.

An Agilent 5975 mass spectrometer (MS) coupled to an Agilent 7820A gas chromatography (GC) system (Santa Clara, CA, USA) was also used to identify the reaction products. For sample preparation, water was evaporated, and the samples were then dissolved in acetonitrile. The separation of the compounds was carried out using a 30-meter-long HP-5MS capillary column (Santa Clara, CA, USA).

## 4. Conclusions

In this work, nanocomposites with uniformly distributed mono- and bimetallic nanoparticles (Ni, Au, and Ni-Au) on a ZnAlO support were developed. These materials were intensively characterized to correlate their structural and physicochemical properties with catalytic performances in BPA photodegradation. The UV-Vis spectrum shows that the presence of nanoparticles extends the light absorption into the visible range, enabling a more efficient utilization of sunlight. The enhanced catalytic performance of the Ni-Au@ZnAlO catalyst can be attributed to its larger active surface area, which facilitates a more effective interaction with BPA. Notably, the Ni-Au@ZnAlO catalyst demonstrated superior photocatalytic performance, achieving up to 95% degradation of Bisphenol A under simulated solar light irradiation. These findings offer promising implications for the efficient removal of persistent organic pollutants.

## Figures and Tables

**Table 1:** Specific surface area and atomic composition of the catalysts determined by XPS analysis.

Catalyst	SSA * (m^2^·g^−1^)	Zn (at.%)	Al (at.%)	Ni (at.%)	Au (at.%)	O (at.%)	Zn^2+^/Al^3+^ Atomic Ratio
ZnAlO	86	36.4	11.0	-	-	52.6	3.3
Ni@ZnAlO	81	37.2	9.7	0.4 (1.36 **)	-	52.8	3.8
Au@ZnAlO	78	33.6	11.4	-	0.12 (1.87 **)	55.0	2.9
Ni-Au@ZnAlO	74	33.1	10.2	1.2	0.10 (0.96 **)	55.4	3.2

* SSA is the specific surface area. ** Quantitative analysis of elements determined by XRD using the Reference Intensity Ratio (RIR) method.

**Table 2:** The H_2_-chemisorption and H_2_-TPR results of the prepared catalysts.

Catalyst	Metallic Surface Area (m^2^·g^−1^)	Particle Size (nm)	Dispersion (%)	Total H_2_ Consumption (µmol·g^−1^)
ZnAlO	-	-	-	-
Ni@ZnAlO	2.03	2.73	41.40	171
Au@ZnAlO	-	-	-	5
Ni-Au@ZnAlO	2.46	1.97	52.21	146

**Table 3:** The photocatalytic parameters of the studied photocatalysts.

Photocatalyst	Rate Constant of BPA Degradation	Conversion of BPA (%)
	k (min^−1^)	
Photolysis (without catalyst)	0.0003	6.64
ZnAlO	0.0011	15.67
Ni@ZnAlO	0.0062	67.38
Au@ZnAlO	0.0124	87.22
Ni-Au@ZnAlO	0.0179	95.05

**Table 4:** Comparison of the literature data for the photocatalytic degradation of bisphenol A over various catalytic systems.

Photocatalyst	Reaction Conditions	Light Source	Irradiation Time	Removal Efficiency (%)	Ref.
Ag/ZnO/ZnAl-LDH	Conc. of BPA = 10 mg·L^−1^ 0.50 g L^−1^ of cat.	120 W LED lamp, λ = 380–780 nm	360 min	80	[67]
TiO_2_@ZnO/SnO_2_	Conc. of BPA = 20 mg·L^−1^ 0.10 g of cat.	300 W Xe lamp UV light	120 min	58	[68]
10 wt% CuBi_2_O_4_/ZnAl-LDH	Conc. of BPA = 20 mg·L^−1^ 0.20 g of cat.	500 W Xe lamp (λ_cut_∼420 nm) visible light	240 min	84	[69]
Ag_2_C_2_O_4_/Ag@GNS/PMS	Conc. of BPA = 10 mg·L^−1^ 0.06 g of cat.	Visible light (λ > 420 nm)	180 min	93	[70]
1.5 wt% Pd/mpg-C_3_N_4_	Conc. of BPA = 20 mg·L^−1^ 1.00 g/L of cat.	350 W Xe lamp Simulated solar light	180 min	93.9	[71]
Ni-Au@ZnAlO	Conc. of BPA = 25 mg·L^−1^ 0.05 g of cat.	150 W Xe short-arc lamp Simulated solar light	180 min	95	This study

## Data Availability

The original contributions presented in the study are included in the article/Supplementary Materials, further inquiries can be directed to the corresponding authors.

## Supplementary Materials

The following supporting information can be downloaded at: https://www.mdpi.com/article/10.3390/molecules30153249/s1, Figure S1. The ATR spectra of: (a) ZnAlO; (b) Ni@ZnAlO; (c) Au@ZnAlO; and (d) Ni-Au@ZnAlO nanocomposites, Figure S2: UV-Vis spectra of mono- (Ni, Au), bimetallic (Ni-Au) nanoparticles, and ZnAl LDH precursor, Figure S3: The XPS characteristic peak of Zn 2p^3/2^ and Zn LMM Auger spectra of (a) ZnAlO, (b) Ni@ZnAlO, (c) Au@ZnAlO, and (d) Ni-Au@ZnAlO catalysts, Figure S4: XPS spectra of Al 2p for (a) ZnAlO, (b) Ni@ZnAlO, (c) Au@ZnAlO, and (d) Ni-Au@ZnAlO catalysts, Table S1: The binding energy for Zn 2p^3/2^ (in eV), the binding energy for Zn LMM Auger (in eV) and the calculated Auger parameter (in eV) over the studied catalysts, Figure S5. Conversion of BPA versus time in the stability cycles for the Ni-Au@ZnAlO catalyst.
