# Supplementary material for: Mono-(Ni, Au) and Bimetallic (Ni-Au) Nanoparticles-Loaded ZnAlO Mixed Oxides as Sunlight-Driven Photocatalysts for Environmental Remediation
Source: Molecules. 2025 Aug 2;30(15):3249. doi: 10.3390/molecules30153249 (PMC12348654; doi:10.3390/molecules30153249)
Supplement: Supplementary file 1 [file molecules-30-03249-s001.zip › molecules-3767257-supplementary.pdf]

Supplementary Materials

Mono-(Ni, Au) and Bimetallic (Ni-Au) Nanoparticles-Loaded ZnAlO Mixed Oxides as Sunlight-Driven Photocatalysts for Environmental Remediation

Monica Pavel [1], Liubovi Cretu [1], Catalin Negrila [2], Daniela C. Culita [1], Anca Vasile [1], Razvan State [1], Ioan Balint [1,*] and Florica Papa [1,*]

[1] "Ilie Murgulescu" Institute of Physical Chemistry of the Romanian Academy, 202 Spl. Independentei, 060021 Bucharest, Romania; mpavel@icf.ro (M.P.); lcretu@icf.ro (L.C.); danaculita@yahoo.co.uk (D.C.C.); avasile@icf.ro (A.V.); rstate@icf.ro (R.S.)

[2] National Institute of Material Physics, P.O. Box MG 7, 077125 Magurele, Romania; catalin.negrila@infim.ro

* Correspondence: ibalint@icf.ro (I.B.); frusu@icf.ro (F.P.)

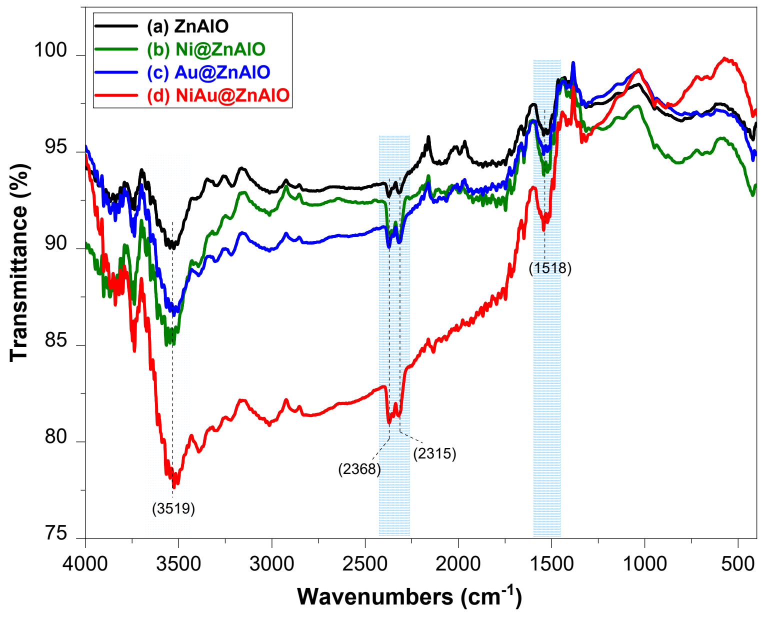

Figure S1. The ATR spectra of: (a) ZnAlO; (b) Ni@ZnAlO; (c) Au@ZnAlO; and (d) Ni-Au@ZnAlO nanocomposites.

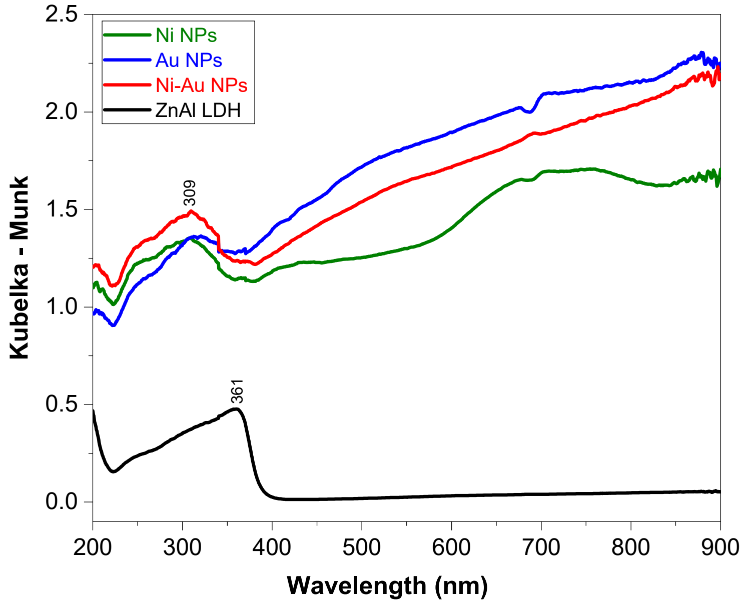

Figure S2. UV-Vis spectra of mono- (Ni, Au), bimetallic (Ni-Au) nanoparticles, and ZnAl LDH precursor.

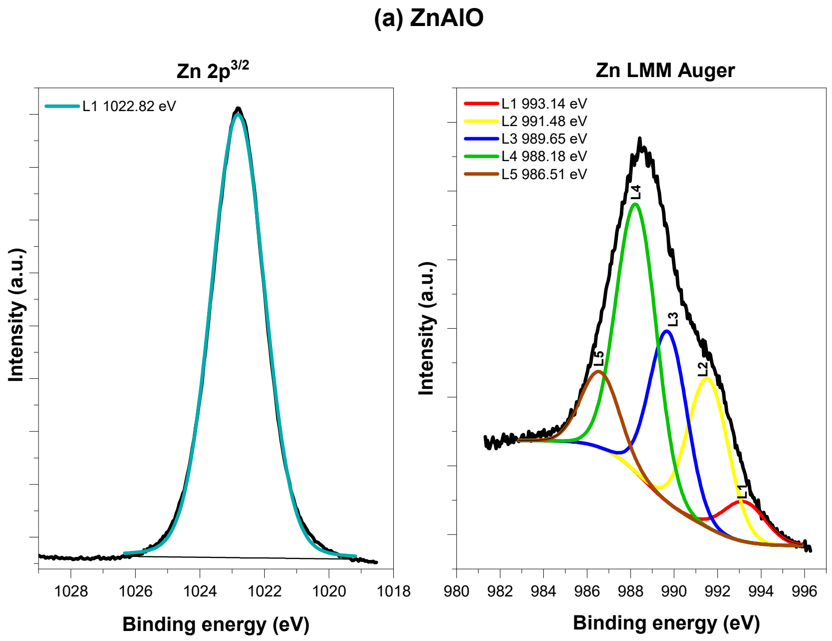

(b) Ni@ZnAlO

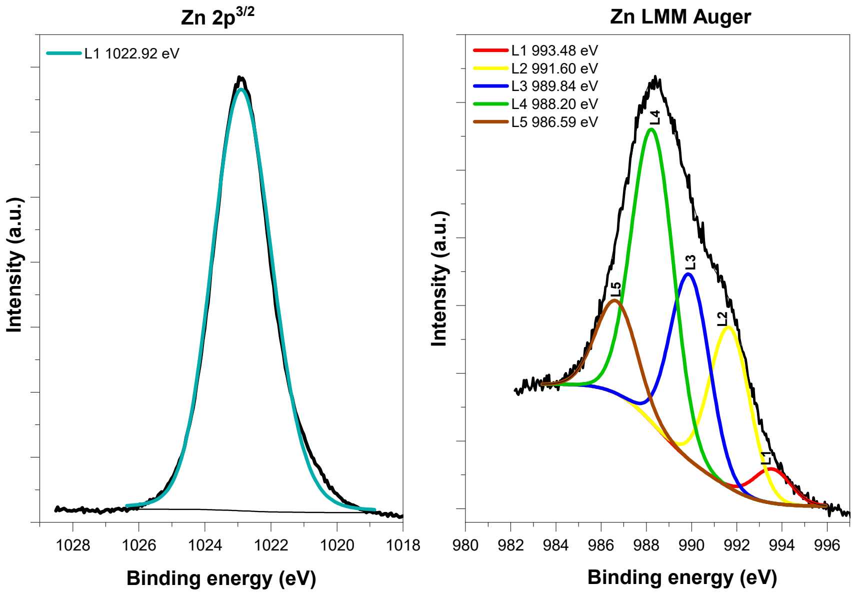

(c) Au@ZnAlO

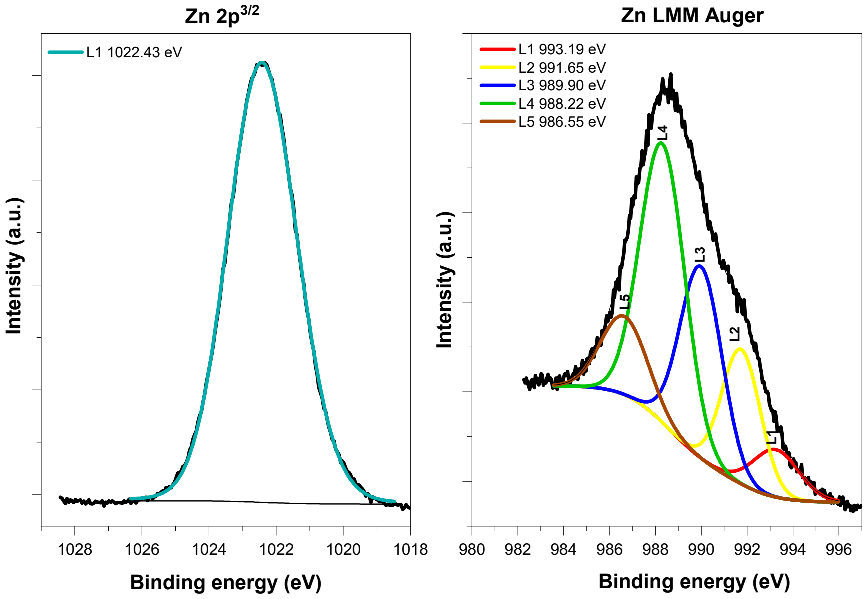

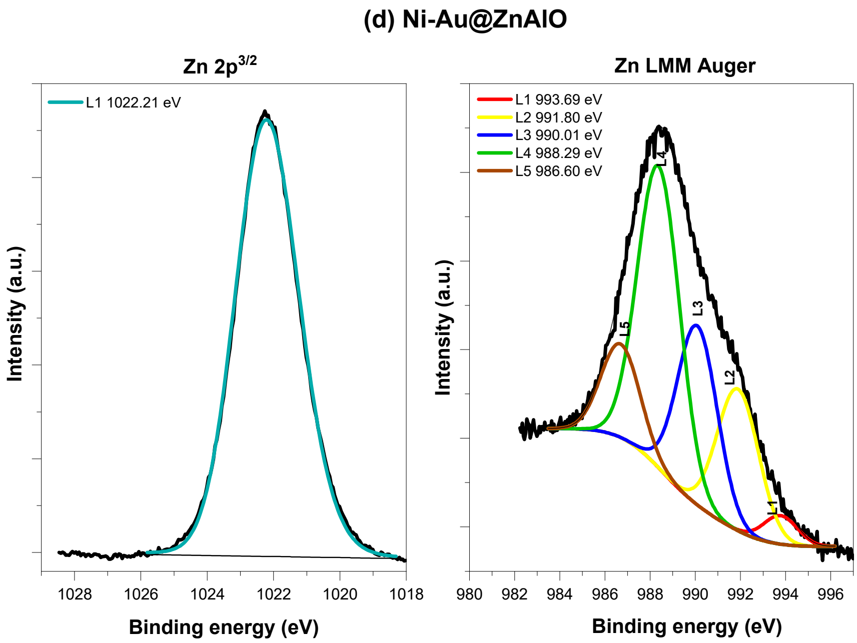

Figure S3. The XPS characteristic peak of Zn $2p^{3/2}$ and Zn LMM Auger spectra of (a) ZnAlO, (b) Ni@ZnAlO, (c) Au@ZnAlO, and (d) Ni-Au@ZnAlO catalysts.

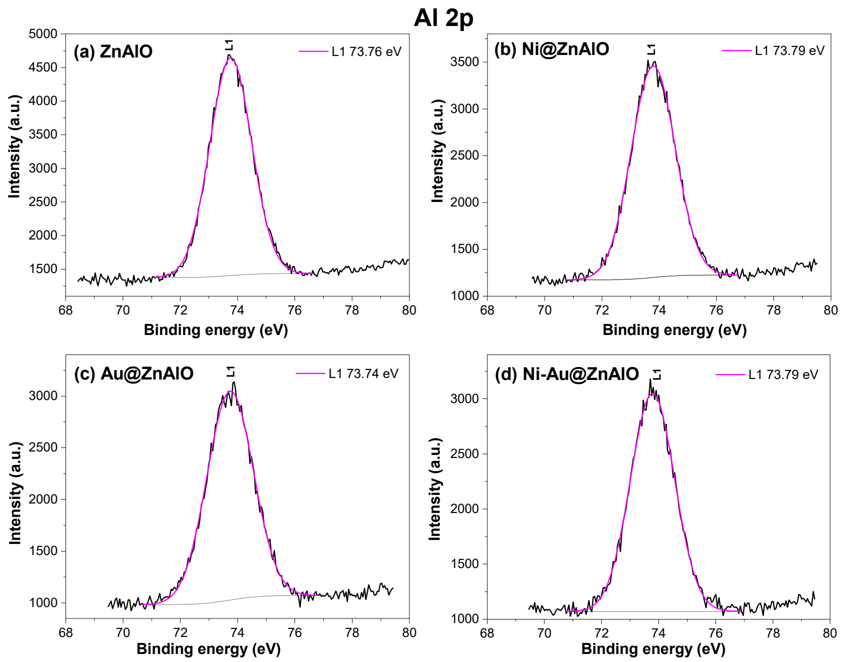

Figure S4. XPS spectra of Al 2p for (a) ZnAlO, (b) Ni@ZnAlO, (c) Au@ZnAlO, and (d) Ni-Au@ZnAlO catalysts

Table S1. The binding energy for Zn $2p^{3/2}$ (in eV), the binding energy for Zn LMM Auger (in eV) and the calculated Auger parameter (in eV) over the studied catalysts.

Catalyst	Zn $2p^{3/2}$	Max. (Zn LMM Auger)	Modified Auger parameter
ZnAlO	1022.82	988.18	2011.00
Ni@ZnAlO	1022.90	988.20	2011.10
Au@ZnAlO	1022.43	988.22	2010.65
Ni-Au@ZnAlO	1022.21	988.29	2010.50

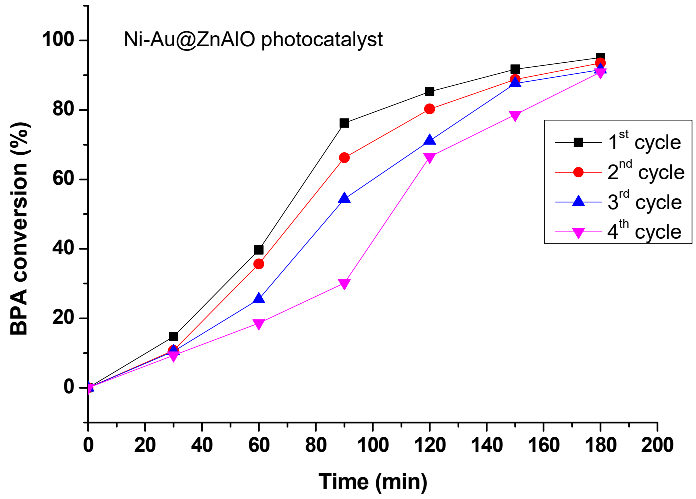

Figure S5. Conversion of BPA versus time in the stability cycles for the Ni-Au@ZnAlO catalyst.
